# Identification and Characterization of Novel Antibody Epitopes on the N2 Neuraminidase

**DOI:** 10.1128/mSphere.00958-20

**Published:** 2021-02-10

**Authors:** Ericka Kirkpatrick Roubidoux, Meagan McMahon, Juan Manuel Carreño, Christina Capuano, Kaijun Jiang, Viviana Simon, Harm van Bakel, Patrick Wilson, Florian Krammer

**Affiliations:** a Department of Microbiology, Icahn School of Medicine at Mount Sinai, New York, New York, USA; b Graduate School of Biomedical Sciences, Icahn School of Medicine at Mount Sinai, New York, New York, USA; c The Global Health and Emerging Pathogens Institute, Icahn School of Medicine at Mount Sinai, New York, New York, USA; d Division of Infectious Diseases, Department of Medicine, Icahn School of Medicine at Mount Sinai, New York, New York, USA; e Department of Genetics and Genomic Sciences, Icahn School of Medicine at Mount Sinai, New York, New York, USA; f Icahn Institute for Data Science and Genomic Technology, Icahn School of Medicine at Mount Sinai, New York, New York, USA; g Department of Medicine, Section of Rheumatology, Gwen Knapp Center for Lupus and Immunology Research, University of Chicago, Chicago, Illinois, USA; University of Kentucky College of Medicine

**Keywords:** N2, epitopes, neuraminidase, influenza, mAb

## Abstract

The influenza virus neuraminidase (NA) is becoming a focus for novel vaccine designs. However, the epitopes of human anti-NA antibodies have been poorly defined. Using a panel of 10 anti-N2 monoclonal antibodies (MAbs) that bind the H3N2 virus A/Switzerland/9715293/2013, we generated five escape mutant viruses. These viruses contained mutations K199E/T, E258K, A272D, and S331N. We found that mutations at K199 and E258 had the largest impact on MAb binding, NA inhibition and neutralization activity. In addition, a natural isolate from the 2017-2018 season was found to contain the E258K mutation and was resistant to numerous antibodies tested. The mutation S331N, was identified in virus passaged in the presence of antibody; however, it had little impact on MAb activity and greatly decreased viral fitness. This information aids in identifying novel human MAb epitopes on the N2 and helps with the detection of antigenically drifted NAs.

**IMPORTANCE** The influenza virus neuraminidase is an emerging target for universal influenza virus vaccines. However, in contrast to influenza virus hemagglutinin, we know little about antibody epitopes and antigenic sites on the neuraminidase. Characterizing and defining these sites is aiding vaccine development and helping to understand antigenic drift of NA.

## INTRODUCTION

Influenza viruses are respiratory pathogens that cause seasonal outbreaks and, occasionally, global pandemics. Annually, influenza viruses cause significant morbidity and mortality (1). Vaccinations against influenza virus are administered seasonally; however, vaccine effectiveness is generally low (20 to 60%) (2). Representative strains to be included in influenza virus vaccines are selected based on the antigenicity of their hemagglutinin (HA), the most abundant glycoprotein on the surface of the virion (3, 4). Antibody responses induced by vaccination generally target the hypervariable, immunodominant head domain of the HA (5–7). Anti-HA antibodies have long been considered the “gold standard” of anti-influenza virus immunity since they can readily neutralize the virus and induce—at least in some animal models—sterilizing immunity. Subsequently, influenza virus vaccines are standardized by HA content, whereas the amount of the second viral glycoprotein, the neuraminidase (NA), in any given formulation is variable (8). However, there is increasing evidence that anti-NA immunity can substantially contribute to protection and should also be standardized in influenza virus vaccines (9–11).

The NA is a sialidase which cleaves terminal sialic acids from N-linked glycans on glycoproteins. The protein is enzymatically active as a homotetramer and has two domains, the head (which contains the active site) and the stalk (12). The NA is primarily involved in viral transmission through the cleavage of decoy receptors in the mucosa, preventing viral aggregation and releasing newly formed virions from infected cells; achieving these functions through its enzymatic activity (13, 14). There are nine subtypes of NA which are organized into group 1 (N1, N4, N5, and N8) and group 2 (N2, N3, N6, N7, and N9) (4). Currently, NA inhibitors are prescribed to aid in reducing influenza disease progression and virus transmission. There are several neuraminidase inhibitors on the market that block the enzymatic activity of the NA. These include Relenza (zanamivir), Tamiflu (oseltamivir), Rapivab (peramivir), and Inavir (laninamivir). Unfortunately, influenza viruses can become resistant to these inhibitors, greatly reducing their efficacy. The mutations E119V, R292K, and N294S have been shown to confer oseltamivir resistance in N2 containing viruses (15).

The NA is immunogenic and antibody responses toward this viral glycoprotein are an independent correlate of protection (8, 16–22). However, these antibodies are infection permissive and prevent viral egress and dissemination through NA inhibition (NAI) activity instead of neutralizing virions prior to infection (11, 19, 23, 24). Typically, the antibodies produced can cross-react with other similar viruses within a subtype; however, they are usually not cross-reactive with other NA subtypes (8, 23, 25).

There have been several studies using MAbs to map antigenic regions of the N2 NA (8, 22, 26–30). Early reports described seven “families” of antigenic regions (29). Later studies identified residues that were critical for MAb binding, NAI, and neutralization activity (8, 22, 26–28, 30). However, with the exception of Chen et al. (8) and Stadlbauer et al. (22), as well as Powell and Pekosz (31), these studies were performed using murine antibodies and were therefore not a true reflection of the epitopes targeted by human MAbs. Here, we used a panel of MAbs from Chen et al. (8) to define novel epitopes on the human N2 from the isolate A/Switzerland/9715293/2013 using escape mutagenesis. We then characterized escape mutant viruses (EMVs) to examine how each escape mutation impacted the panel’s binding, NAI, and neutralization activities. Knowing which residues are primarily targeted by the human antibody response can aid in determining whether novel H3N2 isolates will be antigenically distinct from one another. In addition, an understanding of the anti-N2 antibody response can provide insights for rational vaccine design, which may be critical for future NA containing vaccines.

## RESULTS

### Establishing a panel of anti-N2 monoclonal antibodies.

As stated above, many of the current MAb epitopes identified in literature have been elucidated using murine antibodies (8, 22, 26–30). To complement previous studies, we used several MAbs identified and isolated in Chen et al. (8). For epitope analysis we chose a panel of 10 MAbs: 229-1F06, 229-2E02, 229-2G05, 235-1C02, 235-1E06, 229-2B04, 228-1B03, 229-1G03, 229-1D05, and 229-2C06. Some MAbs, such as 229-2E02 and 229-2B04, had low cross-reactivity; however, several MAbs were able to cross-react with avian N2, as well as with N3 and N9 (8). To test NAI activity, we performed an enzyme-linked lectin assay (ELLA) in the presence of MAb and found that all MAbs, aside from 228-1B03, exhibited NAI activity against the H3N2 virus A/Switzerland/9715293/2013, a previous vaccine strain (Table 1). In addition, all MAbs aside from 228-1B03 efficiently neutralized the virus in plaque reduction neutralization assays (PRNAs) (Table 1). Furthermore, all MAbs aside from 229-1F06 were previously tested for *in vivo* protection. The MAbs with low cross-reactivity mentioned above were not protective, while the more cross-reactive MAbs were 60 to 100% protective (8). We included an array of MAbs with various properties to aid in understanding if particular types of MAbs target specific regions of the NA.

**TABLE 1:** NAI and neutralization activity of the anti-N2 MAb panel

MAb	IC_50_ (μg/ml)	
	NAI	PRNA
229-1F06	0.194	0.274
229-2E02	41.015	5.684
229-2G05	26.31	10.11
235-1C02	0.278	0.035
235-1E06	0.222	0.045
229-2B04	12.605	0.461
228-1B03	–^*a*^	–
229-1G03	0.350	0.034
229-1D05	6.092	0.545
229-2C06	0.232	0.601

a –, No activity.

### Generating N2 MAb escape mutant viruses.

**FIG 1:**
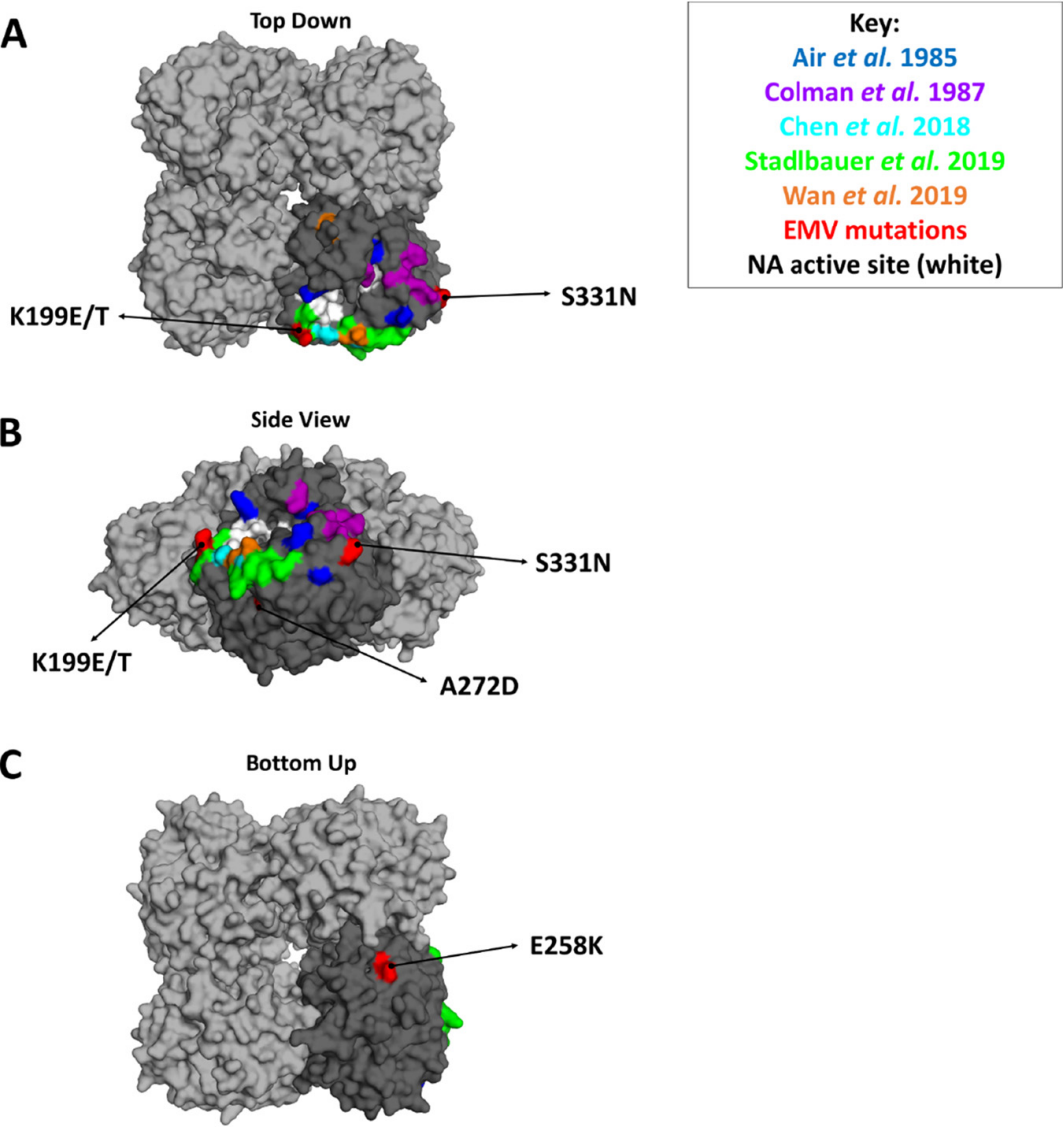
Location of NA escape mutations. (A to C) Top-down (A), side (B), and bottom-up (C) views of the three-dimensional structure of N2 (PDB ID 4GZO [37]). Sites identified by Air et al. (26) are depicted in blue, Colman et al. (27) in purple, Chen et al. (8) in cyan, Stadlbauer et al. (22) in green, and Wan et al. (28) in orange. Escape mutations identified in this study are indicated in red. The NA active site is indicated in white (29). Images were generated using PyMOL v2.1.0.

To determine residues that are important targets of human MAbs, we generated escape mutant virus (EMVs) by serially passaging A/Switzerland/9715293/2013 on Madin-Darby canine kidney (MDCK) cells in the presence of MAbs. We were able to isolate six viruses that contained a mutation in their NA using the MAbs 229-1D05, 235-1C02, 235-1E06, 229-2C06, 229-1F06, and 229-1G03 (Table 2). We found that these EMVs appeared after 2 to 10 passages and identified five distinct NA mutations: K199E/T (229-1D05 and 235-1C02/235-1E06, respectively), E258K (229-2C06), A272D (229-1F06), and S331N (229-1G03) (Table 2 and Fig. 1). Four MAbs in the panel (229-2E02, 229-2G05, 229-2B04, and 228-1B03) did not produce EMVs despite undergoing 10 passages in cell culture.

**TABLE 2:** NA mutations identified via escape mutagenesis^*a*^

Isolate	MAb	No. of passagesto escape	NA mutation	HA mutation(s)
K199E EMV	229-1D05	4	K199E	**Y235S, N262T**
K199T EMV	235-1C02, 235-1E06	2	K199T	S161N, Y239F
E258K EMV	229-2C06	6	E258K	H172R, **Y235S**
A272D EMV	229-1F06	2	A272D	Y235S, N262T
S331N EMV	229-1G03	4	S331N	Y235S, N262T
Irrelevant IgG control virus	KB2	Arbitrarily stopped after 6 passages	No NA mutations	Y235S, N262T
A/New York/PV190/2017	N/A	N/A	S245N, S247T, **E258K**, T267K, N329S, L338F, D339N, I380V, T392I, P468H	ND

a NA mutation, neuraminidase mutation; N/A, not applicable; ND, not determined. Boldfaced mutations are shared with A/New York/PV190/2017 or the irrelevant IgG control virus.

Escape mutations are located in various regions of the protein. The K199E/T mutations are the closest to the NA active site (Fig. 1A and B). E258K is located on the bottom of the NA (Fig. 1C). Both A272D and S331N are located on the side of the NA (Fig. 1A and B). Notably, mutations at E258 and A272 have not been previously reported. The S331N mutation causes the gain of a putative N-linked glycosylation site in the NA. In addition, each EMV contained mutations in several other segments (Table 3). The irrelevant IgG control virus did not contain any NA mutations; however, it did acquire mutations in its PA, HA, M, and NS1 proteins. Once identified, we decided to further characterize the impact of each NA escape mutation on NA antigenicity and viral fitness.

**TABLE 3:** Additional mutations identified in escape mutant viruses^*a*^

Isolate	Mutation(s)				
	PA	HA	NP	M	NS1
K199E EMV		Y235S, N262T	G349R	L109F, R222H	
K199T EMV		S161N, Y239F	D101G, N290D	L109F, R222H	
E258K EMV		H172R, Y235S		L109F, R222H	
A272D EMV		Y235S, N262T		L109F, R222H	
S331N EMV		Y235S, N262T	G102R, N290D	L109F, R222H	
Irrelevant IgG control virus	E623G	Y235S, N262T		L109F	D2N

a Only segments containing mutations are listed. Numbering is from methionine.

### Escape mutations cause changes in MAb binding, neuraminidase inhibition, and neutralization activities.

**FIG 2:**
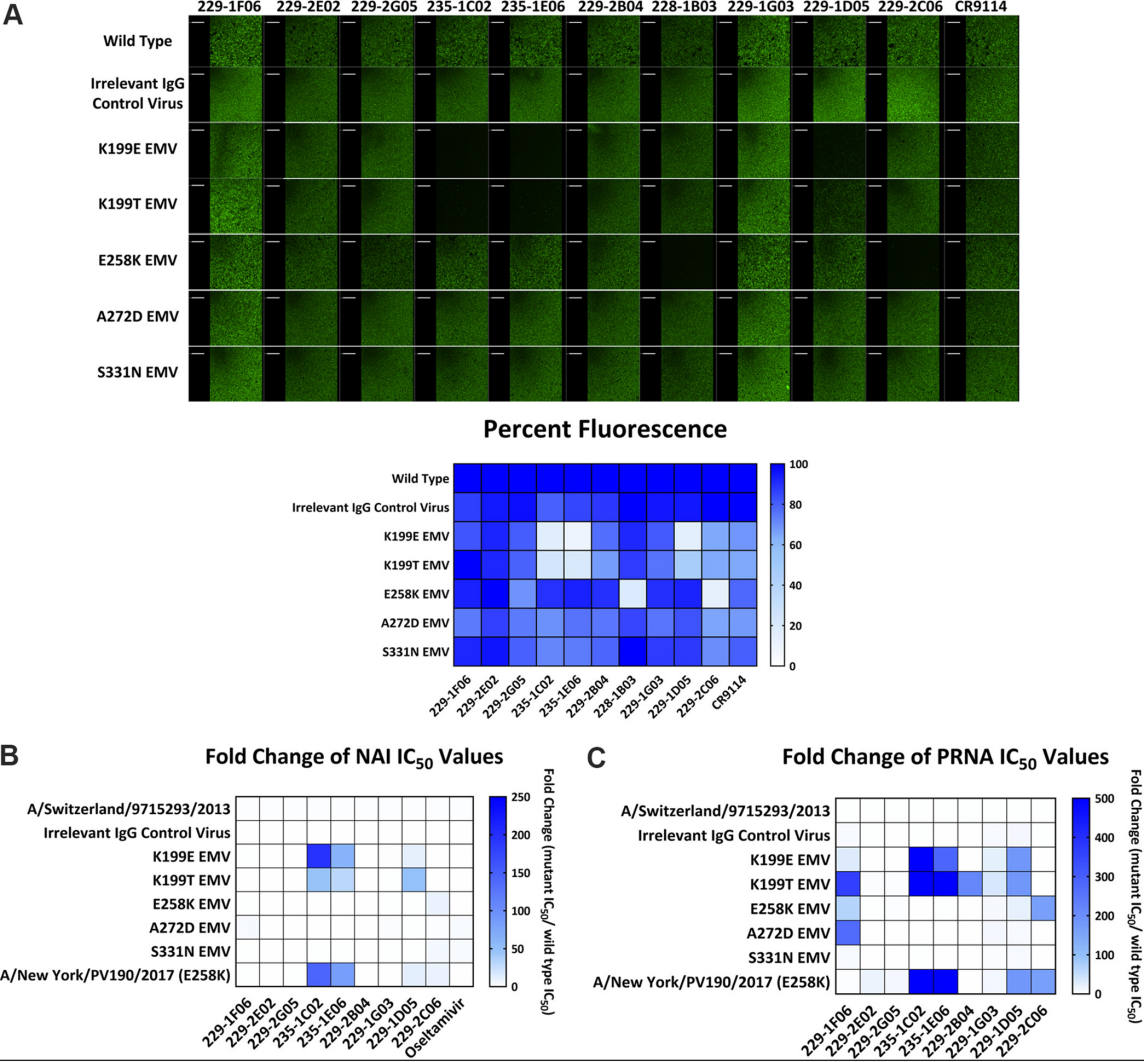
Characterization of escape mutant viruses. (A) Binding of MAbs to wild-type virus and EMVs. Immunofluorescence images for N2 EMVs and the panel of described MAbs. Each image is representative of two independent experiments. The heatmap below illustrates the percent fluorescence compared to the wild type. (B and C) Heatmaps illustrating fold change of NAI IC_50_ (B) and PRNA IC_50_ (C) values with EMVs along the *y* axis and MAbs along the *x* axis. The IC_50_ values were calculated using Prism 7.0, and the fold change was determined by dividing EMV values by wild-type virus values. Darker blue indicates a stronger escape phenotype. NAI assays and PRNAs were conducted in duplicate.

Using our panel of antibodies, we investigated which NA mutations had effects on MAb binding, neutralization and NA inhibition activities. The NA inhibition and neutralization activities of these antibodies against wild-type virus vary (Table 1). We first tested whether the NA mutations had an impact on binding using an immunofluorescent staining assay. We found that the residues K199 and E258 were the most important for MAb binding (Fig. 2A). Notably, MAb 228-1B03, which does not have NAI or neutralizing activity, lost binding to the E258K EMV.

We next investigated how each escape mutation affected both the NAI and neutralization activity of each MAb. We found that the mutations in our EMVs conferred complete escape or resistance to several antibodies in the panel, including MAbs that were unable to produce EMVs (Fig. 2B and Table 4). NAI assays indicated that the K199E mutation caused complete escape from the MAb 235-1C02 and caused resistance to 235-1E06 (62-fold increase in NAI 50% inhibitory concentration [IC_50_]) and 229-1D05 (11-fold increase in NAI IC_50_). The K199T mutation also affected those same MAbs with a 47-fold increase in NAI IC_50_ for 235-1C02, a 30-fold increase in NAI IC_50_ for 235-1E06, and a 48-fold increase in NAI IC_50_ for 229-1D05. The E258K mutation caused complete escape from MAb 229-2C06, but otherwise it did not have much impact on NAI activity. Although the A272D EMV was generated using 229-1F06, we observed only a 4-fold increase in the MAb’s NAI IC_50_ and no resistance against the other MAbs in the panel. The S331N EMV, selected with 229-1G03, became slightly resistant toward 229-2C06 (5-fold increase in NAI IC_50_); however, it did not show resistance toward any other MAbs in the panel. We also identified an isolate circulating in New York City during the 2017-2018 influenza season that contained the same E258K mutation identified through escape mutagenesis, A/New York/PV190/2017 (Table 2). The A/New York/PV190/2017 virus completely escaped 229-2C06 and became resistant to 235-1C02 (144-fold increase in NAI IC_50_), 235-1E06 (85-fold increase in NAI IC_50_) and 229-1D05 (12-fold increase in NAI IC_50_). A summary of NAI IC_50_ values can be found in Table 4.

**TABLE 4:** Summary of NAI IC_50_ values for escape mutant viruses

Virus	MAb NAI IC_50_ (μg/ml)^*a*^									
	229-1F06	229-2E02	229-2G05	235-1C02	235-1E06	229-2B04	229-1G03	229-1D05	229-2C06	Oseltamivir
A/New York/PV190/2017 (E258K)	0.130	0.039	14.380	40.020	18.800*	0.004	0.037	4.355	60.000	0.003
S331N EMV	0.147	4.289	11.780	0.140	0.164	1.183	0.086	0.216	32.530	0.649
A272D EMV	0.756	3.313	7.326	0.047	0.068	2.332	0.343	0.226	8.568	0.668
E258K EMV	0.097	2.274	3.615	0.075	0.082	0.179	0.074	0.167	60.000	0.066
K199T EMV	0.072	0.684	1.350	12.960	6.725	0.054	0.079	16.830	0.360	0.035
K199E EMV	0.103	6.669	11.290	54.56**	13.780	0.201	0.138	3.846	0.887	0.064
Irrelevant IgG control virus	0.032	0.507	0.800	0.021	0.021	0.037	0.015	0.068	0.168	0.020

a The highest MAb concentration tested (60 μg/ml) is listed when complete escape occurred. Boldface values indicate statistically significant changes in IC_50_ values based on technical replicates. *, *P* < 0.0332; **, *P* < 0.0001.

The NA mutations had a broader impact on MAb mediated neutralization compared to NAI activity (Fig. 2C and Table 5). We measured neutralization through PRNAs and found that the K199E mutation resulted in complete escape from the MAbs 235-1C02 and 229-1D05 and resistance to 229-1F06 (30-fold increase in neutralizing IC_50_), 235-1E06 (287-fold increase in neutralizing IC_50_), and 229-1G03 (23-fold increase in neutralizing IC_50_). The K199T mutation had stronger effects on neutralization activity, although the same MAbs were impacted. It caused complete escape from 229-1F06, 235-1C02, 229-2B04, and 229-1D05 along with resistance to 229-1G03 (34-fold increase in neutralizing IC_50_). The E258K EMV exhibited complete escape from 229-2C06, along with resistance to 229-1F06 (66-fold increase in neutralizing IC_50_). The A272D EMV almost completely escaped from 229-1F06 (2,274-fold increase in neutralizing IC_50_). Interestingly, this mutation had a much weaker effect on NAI activity compared to neutralization activity. The S331N mutation had very little effect on MAb neutralization. A/New York/PV190/2017 completely escaped 229-2E02, 229-2G05, 235-1C02, 235-1E06, 229-1D05, and 229-2C06. A summary of the neutralizing IC_50_ values for each MAb against the EMVs can be found in Table 5. These results indicate that both the K199 and the E258 residues are critical for a number of MAbs.

**TABLE 5:** Summary of PRNA IC_50_ values for escape mutant viruses

Virus	MAb NAI IC_50_ (μg/ml)^*a*^								
	229-1F06	229-2E02	229-2G05	235-1C02	235-1E06	229-2B04	229-1G03	229-1D05	229-2C06
A/New York/PV190/2017 (E258K)	0.756	100	100	100**	100*	0.226	0.353	100	100
S331N EMV	1.887	0.501	0.443	0.032	0.034	0.063	0.150	3.988	0.148
A272D EMV	75.260	0.415	0.508	0.029	0.032	0.086	0.324	4.167	0.113
E258K EMV	18.160	5.433	26.720	0.030	0.062	0.154	0.296	11.830	1,000
K199T EMV	100	23.010	22.490	100**	53.710	1,000	1.189	1,000	0.801
K199E EMV	8.161	0.777	0.813	100**	12.970	0.164	0.800	100	0.173
Irrelevant IgG control virus	1.924	5.947	1.318	0.039	0.039	0.234	0.251	5.302	0.868

a The highest MAb concentration tested (100 μg/ml) is listed when we observed complete escape. Boldface values indicate statistically significant changes in IC_50_ values based on technical replicates. *, *P* < 0.0332; **, *P* < 0.0021.

### Escape mutations have effects on virus growth *in vitro*.

**FIG 3:**
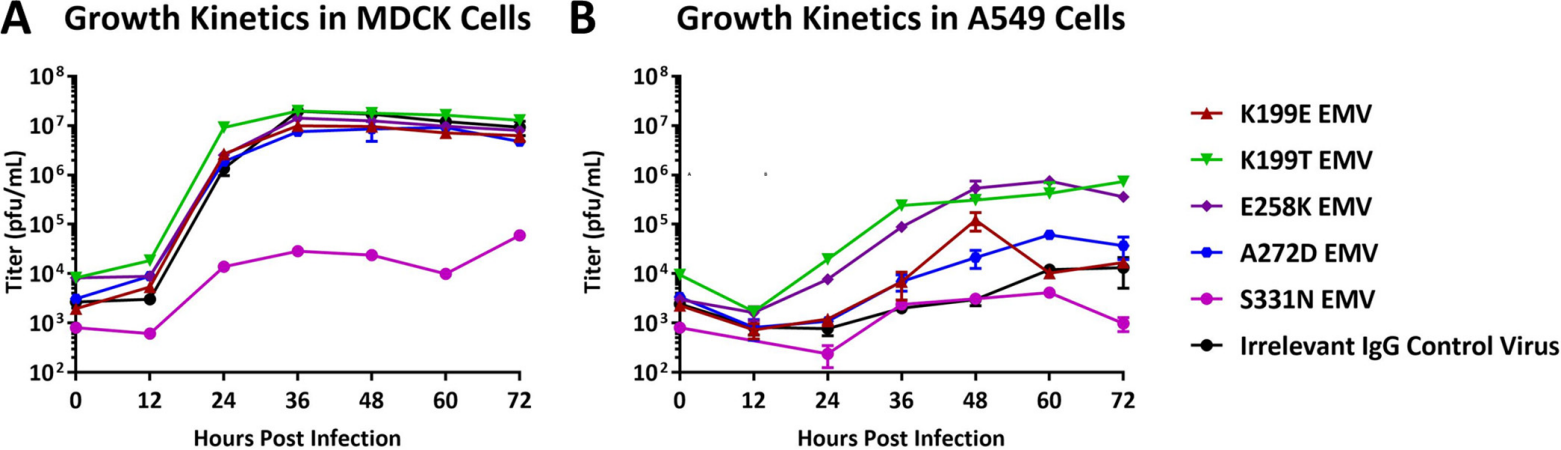
Growth kinetics of escape mutant viruses. (A and B) Titers for each EMV at selected time points (every 12 h postinfection) in MDCK (A) and A549 (B) cells. Growth curves were determined in duplicates. Titers for T0 were determined by using the initial inoculum. Error bars indicate the standard deviations from the mean.

A majority of the identified escape mutations have not been identified in natural isolates. Therefore, we assessed how these mutations alter virus fitness *in vitro* to determine the impact of each NA escape mutation on viral replication. Each EMV was generated using MDCK cells; however, we performed growth curve assays using both MDCK cells and the human lung epithelial cell line, A549. All EMVs aside from the S331N EMV grew to similar, high levels in MDCK cells with titers peaking around 1 × 10^7^ PFU/ml (Fig. 3A). However, the growth kinetics of each EMV in A549 cells varied (Fig. 3B). Both the K199T and the E258K EMVs grew to higher titers than the irrelevant IgG control virus (with peak titers of 3.12 × 10^5^ and 5.3 × 10^5^ PFU/ml, respectively, at 48 h postinfection). The K199E and A272D EMVs showed intermediate growth, reaching 1.2 × 10^5^ PFU/ml and 2.1 × 10^4^ PFU/ml at 48 h postinfection, respectively. Both the irrelevant IgG control virus and the S331N virus grew poorly compared to the other EMVs. The irrelevant IgG control virus reached titers of 1.2 × 10^4^ PFU/ml, although this peak was at 60 h postinfection compared to 48 h postinfection for the other EMVs. The S331N EMV did not replicate above 1 × 10^3^ PFU/ml. Interestingly, the EMVs containing mutations found in nature (K199E/T and E258K) had increased fitness in human lung epithelial cells compared to the irrelevant IgG control virus. However, we noticed that four of the six EMVs (K199E, A272D, S331N, and the irrelevant IgG control virus) contained the HA mutation N262T, which causes a loss of an N-linked glycosylation site (Table 2). The K199T and E258K EMVs did not contain this HA mutation and also grew to the highest titers in A549 cells, suggesting that N262T is altering viral fitness. Due to poor replication kinetics and low infectivity of recent H3N2 viruses in mice, we were unable to complete *in vivo* fitness studies (32).

## DISCUSSION

The epitopes for human anti-HA MAbs have been studied extensively. However, the N2 antigenic sites for human anti-NA MAbs have not been fully defined. To accurately designate MAb epitopes on the N2, we began by determining whether the mutations found using our panel of antibodies overlapped with those previously identified. We found that position 199 was identified as an important MAb interacting residue by Colman et al. (29), Gulati et al. (30), and Stadlbauer et al. (22).

In our study, we found that 199 was critical for MAbs 229-1D05 and 235-1C02. This residue is located in a loop that “hangs” over the opening to the enzymatic site. Additional studies have confirmed our results and also identified 235-1E06 as another MAb that targets 199 (28–30). Mutations A272D and S331N have not been identified in other studies. Our A272D EMV showed resistance toward the MAb 229-1F06 in neutralization assays; however, it retained NAI activity. In addition, the S331N EMV did not become resistant to any MAbs in the panel. Despite 331 being identified by Colman et al. (27) as a variable residue, our S331N EMV did not behave as a proper escape mutant. These data suggest that mutations such as A272D and S331N may not be sufficient for complete escape from MAb NAI activity. We also identified the escape mutation at residue 258, which is located on the bottom of the NA. This mutation was important for resistance to 229-1F06 and 229-2C06. In addition, E258K was present in A/New York/PV190/2017 and allowed the virus to completely escape the MAb 229-2C06 (which selected the E258K EMV). This makes E258 a unique target for human antibodies, suggesting that changes at 258 and elsewhere are occurring to avoid antibody responses. The identification of these important sites for MAb binding can aid in the detection of antigenically distinct N2 proteins. In particular, residue 199 seems to be the most critical for binding, NAI and neutralization activity of several MAbs (28–30). The emergence of mutations in these epitopes could indicate antigenic drift is occurring in viral populations.

In addition, we noticed that escape mutations had a stronger impact on MAb neutralization activity compared to NAI activity. This may be attributed to the mechanisms of neutralization for anti-NA MAbs, which rely on inhibiting NA activity and preventing viral spread rather than preventing initial infection (12, 13). NAI activity can be achieved by either directly or indirectly blocking the NA enzymatic site from cleaving sialic acid. However, neutralization activity (if determined through PRNAs) can only be achieved by MAbs with NAI activity that is so potent it prevents viral spread before detectable plaques can form. Our data show that subtle changes in NAI activity for a single MAb may cause much larger changes in neutralization activity. For example, the A272D EMV escaped neutralization by the MAb 229-1F06; however, there was only a 4-fold increase in the NAI IC_50_. We did not observe any MAbs that lost NAI activity but retained neutralization activity. Of note, the MAbs with the weakest NAI activity—229-2E02, 229-2G05, 229-2B04, and 228-1B03—did not generate EMVs. For future studies, it would be important to determine how well human sera can inhibit our identified escape mutant viruses, along with new H3N2 isolates, to fully determine the extent of effects of NA mutations on overall anti-NA responses in the human host. By monitoring drift in both HA and NA, we could potentially greatly increase vaccine efficacy and reduce the overall morbidity and mortality caused by influenza virus.

## MATERIALS AND METHODS

### Cells and viruses.

A549 (ATCC CCL-185) and MDCK (ATCC CCL-34) cells were obtained from the American Type Culture Collection (ATCC) and propagated in 1× Dulbecco's modified Eagle's medium (DMEM) supplemented with 10% heat-inactivated fetal bovine serum (Sigma-Aldrich), 1 U/ml penicillin–1 μg/ml streptomycin solution (Gibco), and 10 mM HEPES {2-[4-(2-hydroxyethyl)piperazin-1-yl]ethanesulfonic acid} (Gibco). Cells were kept at 37°C with 5% CO_2_. A/Switzerland/9715293/2013 was obtained from the Influenza Reagent Resource (FR-1366) and grown in 10-day-old specific-pathogen-free (SPF) embryonated chicken eggs (Charles River Laboratories) at 33°C for 3 days (33).

### Antibodies.

Plasmids encoding each antibody were provided by Patrick Wilson from the University of Chicago. The isolation and reactivity of each antibody has been previously described (8). Antibodies were produced by cotransfection of heavy and light chain plasmids using an ExpiFectamine 293 transfection kit according to manufacturer’s instructions (Thermo Fisher). Briefly, heavy- and light-chain plasmids were mixed with ExpiFectamine and incubated for 15 min in Opti-MEM (Gibco). HEK293F cells (Thermo Fisher) were diluted to 7 × 10^7^ cells in 30 ml of Expi293 expression medium (Thermo Fisher). The transfection mix was then added to cells, followed by incubation for 7 days at 37°C with 8% CO_2_ with shaking. All antibodies were purified using gravity flow columns packed with protein G-sepharose. They were eluted into a 50-ml Falcon tube with 5 ml of 2 M Tris (pH 10) and concentrated using an Amicon Ultra 30-kDa filter unit (Millipore) (34).

### Plaque assays.

Virus titers were determined using a standard influenza virus plaque assay. MDCK cells were plated at 8 × 10^5^ cells per ml in a 12-well cell culture plate and incubated overnight at 37°C with 5% CO_2_. The following day, allantoic fluid or cell culture supernatant was diluted 1:10 six times using 1× minimal essential medium (MEM; 10% 10× MEM [Gibco], 2 mM l-glutamine [Gibco], 0.1% sodium bicarbonate [Gibco], 10 mM HEPES, 1% 100 U/ml penicillin–100 μg/ml streptomycin solution [Gibco], and 0.2% bovine serum albumin [BSA]). The MDCK cells were washed one time using 1× phosphate-buffered saline (PBS) and then infected with 200-μl portions of each virus dilution. The cells were then incubated at 33°C with 5% CO_2_ for 40 min (the plates were rocked every 10 min). After 40 min, the virus dilutions were aspirated and immediately replaced with an agarose overlay containing 2× MEM, 0.1% diethylaminoethyl (DEAE)-dextran, 1 μg/ml tolylsulfonyl phenylalanyl chloromethyl ketone (TPCK)-treated trypsin, and 0.64% Oxoid agarose. The plates were incubated for 3 days at 33°C with 5% CO_2_. To quantify virus titers, plates were fixed using 3.7% paraformaldehyde (PFA) overnight at 4°C. The overlay was then removed, and the cells were stained with a solution of 20% methanol containing 0.5% crystal violet powder.

### Immunofluorescence.

MDCK cells were plated in a 96-well cell culture plate at 3 × 10^4^ cells/well and incubated overnight at 37°C with 5% CO_2_. The following day, the viruses were diluted to a multiplicity of infection (MOI) of 5 in 1× MEM. Cells were washed with 1× PBS and then infected with 100 μl of diluted virus. The plates were then incubated for 18 h at 33°C with 5% CO_2_. The following day, the cells were fixed using 3.7% PFA at 200 μl per well. For immunofluorescence staining, the PFA was first aspirated from cells and then replaced with 200 μl of blocking solution containing 3% milk (American Bio) diluted in 1× PBS, followed by incubation for 1 h at room temperature. The blocking solution was removed and replaced with 1% milk. The primary antibodies were diluted to 300 μg in 1× PBS and then added at a 1:10 dilution to the 1% milk for a final concentration of 30 μg per well. Each plate was then incubated for 1 h at room temperature with shaking. The primary antibodies were then aspirated, and the plate was washed three times using 1× PBS. The secondary antibody Alexa Fluor 488-goat anti-human IgG(H+L) (Invitrogen) was diluted 1:500 in 1% milk and added to the plate at 100 μl/well. The plate was then incubated for 1 h at room temperature in the dark with shaking. Finally, the secondary antibody was aspirated, and the plate was washed again three times using 1× PBS. A final 50 μl of 1× PBS was added to each well to prevent the cells from drying out. We used a Celigo S adherent cell cytometer (Nexcelom Bioscience) with the two-channel “Target 1 + 2” (merge) setting to visualize the immunofluorescence. Exposure time, gain and focus (set using image-based auto focus with the 488-nm signal as the target) were automatically determined by the machine. Fluorescence was calculated using the default analysis settings, and the percent fluorescence was determined based on the wild-type signal. The images are representative of two independent immunofluorescence assays.

### Escape mutagenesis.

EMVs were generated using wild-type A/Switzerland/9715293/2013. First, virus was diluted to an MOI of 0.01 in 1× MEM supplemented with 1 μg/ml of TPCK-treated trypsin. Antibodies were diluted to 0.25× IC_50_ and added to the diluted virus (for 1.5 ml/well). The virus-MAb mixture was incubated for 1 h at room temperature before being added to a confluent monolayer of MDCK cells. The cells were incubated for 3 days at 33°C with 5% CO_2_. The cell culture supernatant was then collected spun at 13,000 × *g* for 5 min to pellet the cells. Aliquots were stored at −80°C. For subsequent passages, cell culture supernatant was diluted 1:10 in 500 μl of 1× MEM supplemented with 1 μg/ml of TPCK-treated trypsin and added to a confluent monolayer of MDCK cells. This was incubated at 33°C with 5% CO_2_ for 40 min. Then an additional 1 ml of 1× MEM with 1 μg/ml of TPCK-treated trypsin and 0.5× IC_50_ of MAb was added, and the plates were incubated at 33°C for 3 days. At each passage, the amount of MAb was doubled. Virus was passaged with a MAb up to 128× IC_50_. Each passage was screened for the presence of EMVs using a plaque assay that contained >128× IC_50_ of antibody in the overlay. Individual plaques were injected into 10-day-old SPF eggs and propagated as described above.

### RNA extractions and deep sequencing.

The E.Z.N.A viral RNA extraction kit (Omega Bio-Tek) was used for RNA extractions according to the manufacturer’s instructions. Isolated RNA was stored at −80°C until sequencing. Using the MiSeq v2, 300-cycle reagent kit (Illumina), next-generation sequencing was performed, and the genome was assembled using a pipeline developed at the Icahn School of Medicine at Mount Sinai (35). Full-length sequences were aligned to the deep sequenced segments of wild-type A/Switzerland/9715293/2013 to identify point mutations using MUSCLE in MEGA 7.0 (36).

### Enzyme-linked lectin assay (ELLA).

To determine NA activity for each virus, flat-bottom Immulon 4HBX microtiter plates (Thermo Scientific) were coated with 100 μl/well with 25 μg/ml of fetuin (Sigma) diluted in 1× PBS and incubated overnight at 4°C. The following day, virus was serially diluted (3-fold) in sample dilutant buffer (1× PBS with 0.9 mM CaCl_2_, 0.5 mM MgCl_2_ 1% bovine serum albumin (BSA), and 0.5% Tween 20) in a sterile 96-well plate. An additional volume of sample dilutant was then added at a 1:1 ratio. The diluted virus was then incubated for 1 h at room temperature, shaking. The fetuin-coated plates were then washed three times 220 μl of PBS containing 0.1% Tween 20 (PBS-T) per well using an AquaMax 3000 automated plate washer before virus dilutions were transferred to the plate. The samples were then incubated at 33°C for 18 h (overnight). The plates were washed six times with PBS-T, and then 100 μl of peroxidase-conjugated peanut agglutinin (PNA; Sigma) was added at 5 μg/ml, and the plates were incubated for 2 h at room temperature in the dark. PNA was diluted in conjugate dilutant buffer (1× PBS with 0.9 mM CaCl_2_, 0.5 mM MgCl_2_, and 1% BSA). The PNA was removed, and the plates were washed three times with PBS-T. SigmaFast *o*-phenylenediamine dihydrochloride (OPD; Sigma) was diluted in water, added at 100 μl per well, and incubated for 7 min at room temperature. Development was stopped by the addition of 50 μl of 3 M hydrochloric acid, and the absorbance at 490 nm was read using a Synergy H1 hybrid multimode microplate reader (Bio-Tek). Prism 7.0 was used to determine the effective concentration of each virus that would yield detectable NA activity. Each ELLA was performed in triplicate.

### Neuraminidase inhibition assay.

To determine the IC_50_ of each MAb, flat-bottom Immulon 4HBX microtiter plates (Thermo Scientific) were again coated with 100 μl/well of 25 μg/ml fetuin (Sigma) diluted in 1× PBS, and incubated overnight at 4°C. Antibodies were diluted to 120 μg/ml in sample dilutant buffer and then serially diluted 1:3 in a sterile 96-well plate. Virus was diluted to its calculated effective concentration, added to the MAb dilutions at a 1:1 ratio, and incubated for 1 h at room temperature, shaking. Fetuin-coated plates were washed three times with PBS-T, and the virus-MAb dilutions were added. The assay was then performed according to the ELLA procedure above. IC_50_ values were determined using Prism 7.0. NAI assays were completed in duplicates.

### Plaque reduction neutralization assays.

Neutralization IC_50_ values were determined using PRNAs. First, MDCK cells were seeded at 8 × 10^5^ cells/ml onto 12-well plates. The following day, MAbs were diluted to 100 μg/ml in 300 μl of 1× MEM and then serially diluted 1:5 in a 24-well plate to a final concentration of 0.032 μg/ml in 1× MEM. Virus was diluted to 1 × 10^3^ PFU and added to each of the antibody dilutions (50 μl/well). The virus-MAb mixture was incubated at room temperature for 1 h, shaking. The MDCK cells were then washed one time with 1× PBS and immediately infected with 200 μl of the virus-MAb mixture and incubated at 33°C with 5% CO_2_, with the plates rocked every 10 min. In the meantime, the overlay was prepared by diluting MAbs to 100 μg/ml in 625 μl of 2× MEM and then serially diluted 1:5. Then, a mixture of 1× DEAE-dextrane and 1 μg/ml TPCK-treated trypsin in sterile water for injection (Gibco) was added at 180 μl per well. After the 40 min, the inoculum was aspirated (three wells at a time) and immediately replaced by the overlay mixture containing 360 μl of 2% Oxoid agarose so that the MAb concentration within the agarose matched the same MAb concentration of the inoculum. The plates were incubated at 33°C with 5% CO_2_ for 3 days and then fixed with 3.7% PFA overnight at 4°C. The overlay was removed, and the cells were stained as described above. PRNAs were conducted in duplicates.

### Growth kinetics.

Sterile 24-well plates were seeded with MDCK or A549 cells at 4 × 10^5^ cells/well and incubated overnight at 37°C with 5% CO_2_. The following day, the viruses were diluted to an MOI of 0.01 (5 × 10^3^ PFU/well) in 1× MEM supplemented with 0.2 μg/ml TPCK-treated trypsin. The cells were washed with 1× PBS before being infected with virus. An aliquot of this initial virus dilution was kept and used to determine the initial titer of virus at infection. Viruses were incubated for 72 h at 33°C with 5% CO_2_. Cell culture supernatant was sampled every 12 h and frozen at −20°C until virus titers were determined using plaque assays. Each experiment was performed in duplicate.
